# Editorial: Kallikrein-kinin system: insights into a multifunctional system

**DOI:** 10.3389/fphys.2023.1305981

**Published:** 2023-10-16

**Authors:** Guacyara Motta, Luiz Juliano, Zia Shariat-Madar

**Affiliations:** ^1^ Departamento de Bioquímica, Escola Paulista de Medicina, Universidade Federal de São Paulo, São Paulo, Brazil; ^2^ Departamento de Biofísica, Escola Paulista de Medicina, Universidade Federal de São Paulo, São Paulo, Brazil; ^3^ Division of Pharmacology, University of Mississippi, Oxford, MS, United States

**Keywords:** inflammation, blood coagulation, C1-INH, plasminogen, kallikreins, *Plasmodium falciparum* (Pf) malaria

**FIGURE 1:**
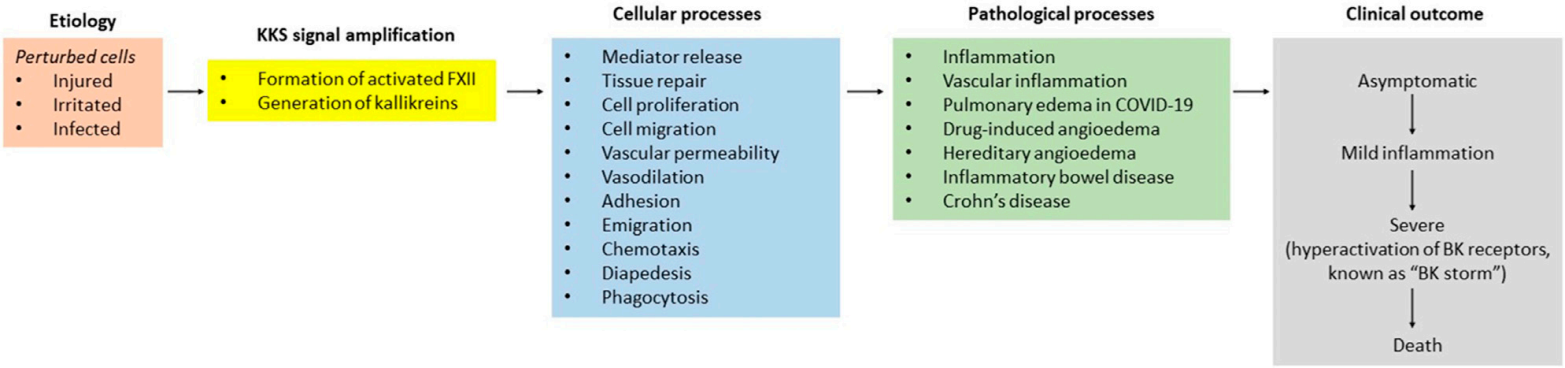
Perturbed cells and the KKS responses to them can be divided into a series of stage.

The plasma kallikrein-kinin system (KKS, known as the contact system) comprises three members: high molecular weight kininogen (HK), prekallikrein (PK), and factor XII. Its signaling is quite simple, with a few components. Injured, irritated, or microorganism-infected cells require careful orchestration of four primary responses: coagulation, inflammation, complement, and immune (Figure 1). The evidence indicates that signaling within and between the molecules that control these events allows for their coordination and ensures the activation of PK to kallikrein (PKa) and FXII to activated FXII (FXIIa).

PKa directly liberates bradykinin (BK) from HK and acts on several substrates, including FXII. While FXII is considered a vital component for the functioning of the intrinsic coagulation cascade, the resulting elevated FXIIa holds a minor role in generating thrombin through the conversion of factor XI (FXI) into the active serine protease FXIa (activated factor XI). Since the thrombotic risk does not increase in hereditary angioedema (HAE) with C1-esterase inhibitor (C1-INH) deficient patients with an ongoing KKS activation (Konings et al., 2013), the generation of thrombin by FXIIa may be necessary for inflammatory exudate (Figure 1). Because formed thrombin can potentially amplify inflammation via both direct and indirect mechanisms (Chen and Dorling, 2009), affecting endothelial cells, vascular smooth muscle cells, monocytes, and platelets (Popović et al., 2012). Consequently, the characterization of FXIIa-mediated generation of thrombin is highly significant in developing an effective treatment for vascular inflammation, particularly severe HAE.

The exact mechanism underlying the activation of KKS and the coordination of this process following wounding remains uncertain. Multiple positive- and negative-feedback loops ensure that KKS is fully committed to wound healing under pathophysiological conditions. For instance, while HK plays a pivotal role in the modulation of immune responses (Cagliani et al., 2013; Oehmcke-Hecht and Köhler, 2018), the C1-INH, a biochemical safety switch, suspends PKa-mediated generation of BK (Curd et al., 1980) to prevent and arrest angioedema via B_2_ receptors (Figure 1). To underscore the significant role of KKS in the blood of humans and animals worldwide, the activation of the KKS concerns to cells, blood, or other body tissues.

Notably, hyperactivation of KKS is observed in severe HAE patients (Bork et al., 2012) and the bronchoalveolar lavage fluid from coronavirus disease 2019 (COVID-19) patients (Garvin et al., 2020), which leads to death. Reduced C1-inhibitor and elevated PKa activities accelerate recurrent episodes of soft-tissue edema via BK. This finding has potential implications in our quest to find treatments that improve inflammatory phases of healing in patients with HAE, patients with acute or chronic inflammatory bowel disease (IBD) (Wang et al., 2018), patients with COVID-19 (da Silva et al., 2022).

This collection of articles in this Research Topic of Frontiers in Physiology features recent studies in KKS, that show both plasma KKS and tissue KKS pathways are well-studied from a molecular point of view. However, we often lack “operational” understanding that would enable us to recognize that KKS pathways are governed by a complex network of molecular interactions involving yet uncharacterized molecules or biochemical functions. This collection of articles addresses cell behavior correlates with BK-mediated B_2_ activation dynamics even though the different perturbations enacted by various blood coagulation proteases, cysteine proteases, or perturbed cell surface are likely to liberate BK from HK.

The article by Motta et al. points out that the plasma KKS and tissue KKS pathways relay many signals for homeostasis in animals, from humans to marine species. This team of scientists thoroughly reviewed the structure and function of KKS. In humans, plasma KKS and tissue KKS pathways are the principal signaling mechanisms for producing a wide array of kinins. The plasma KKS also activates several coagulation protein zymogens in a cascade manner to generate thrombin. Not only kinins and thrombin are critical to inflammation and coagulation, they also stimulate cell proliferation, differentiation, cell migration, and endothelial progenitor cell mobility and function through their respective receptors. Predictably, mutations that increase BK-forming pathway activity affect these processes and can induce vascular leakage and enhanced vasodilation.

Hintze et al. identified another effector protein that contributes to at least a subset of the BK-forming pathway in addition to the principal components of the path. The presented study describes that certain HAE patients, with plasminogen (Plg) mutation p.K330E, offer genetic insights and potential pathways for generating BK from HK. Patients with Plg mutation suffer from angioedema. Plasmin is well-known to be a weak protease downstream of the KKS that is capable of liberating BK from HK. This team shows increased HK cleavage activity in mutant plasminogen compared to wild-type plasminogen. This finding suggests that the B_2_ receptor antagonists may be most effective in people with HAE-causing plasminogen mutation p.K330E. Hintze et al. also present a new approach to HAE-Plg treatment to improve HAE control.

Shamanaev et al. critically reviewed several possible mechanisms of the action of the FXIIa-Lys/Arg309 and p.K330E variants. They outline the potential for the mechanisms underlying the effects of these variants and contrast this with C1-INH-HAE at *in-vitro* and *in-vivo* studies.

Pinheiro et al. discuss the past and current trends of KKS research and highlight the importance of studying the activation pathway and signaling molecules associated with regulating the KKS pathway in cerebral malaria pathogenesis. The KKS pathway generates BK, but its activation is different. The article reviews the activation of KKS via *Plasmodium falciparum* infected red blood cells (iRBCs), and their implication on physiology and pathology. Lastly, while the unique liberation of BK in cerebral malaria is a paramount finding for scientists investigating the structure and function of the components of KKS and their role in disease, Pinheiro et al. also discuss the development and benefits of therapies using kinin inhibitors.

In summary, the articles in this Research Topic focus on KKS in the spectrum of inflammatory disorders and coagulation disorders compared to biological norms. While the plasma KKS has the potential to promote blood clot formation, it also has the potential to cause inflammatory diseases due to its inflammatory mediator. These articles showed that these actions of KKS become exacerbated by various genetic factors, infectious diseases, chronic diseases, and appear to become more pronounced with age. Undoubtedly, a considerable amount of work is still required to explore the importance of KKS in various physiological and pathological conditions to get a complete understanding of their role in health and disease.
